# Drift Diving by Hooded Seals (*Cystophora cristata*) in the Northwest Atlantic Ocean

**DOI:** 10.1371/journal.pone.0103072

**Published:** 2014-07-22

**Authors:** Julie M. Andersen, Garry B. Stenson, Mette Skern-Maurizen, Yolanda F. Wiersma, Aqqalu Rosing-Asvid, Mike O. Hammill, Lars Boehme

**Affiliations:** 1 Department of Biology, Memorial University of Newfoundland, St. John's, Canada; 2 Science Branch, Department of Fisheries and Oceans, Northwest Atlantic Fisheries Centre, St. John's, Canada; 3 Marine Mammal Research Department, Institute of Marine Research, Bergen, Norway; 4 Greenland Institute of Natural Resources, Nuuk, Greenland; 5 Science Branch, Department of Fisheries and Oceans, Institute du Maurice Lamontang, Mont Joli, Quebec, Canada; 6 Sea Mammal Research Unit, Scottish Oceans Institute, University of St. Andrews, St. Andrews, Fife, United Kingdom; The Evergreen State College, United States of America

## Abstract

Many pinniped species perform a specific dive type, referred to as a ‘drift dive’, where they drift passively through the water column. This dive type has been suggested to function as a resting/sleeping or food processing dive, and can be used as an indication of feeding success by calculating the daily change in vertical drift rates over time, which reflects the relative fluctuations in buoyancy of the animal as the proportion of lipids in the body change. Northwest Atlantic hooded seals perform drift dives at regular intervals throughout their annual migration across the Northwest Atlantic Ocean. We found that the daily change in drift rate varied with geographic location and the time of year and that this differed between sexes. Positive changes in buoyancy (reflecting increased lipid stores) were evident throughout their migration range and although overlapping somewhat, they were not statistically associated with high use areas as indicated by First Passage Time (FPT). Differences in the seasonal fluctuations of buoyancy between males and females suggest that they experience a difference in patterns of energy gain and loss during winter and spring, associated with breeding. The fluctuations in buoyancy around the moulting period were similar between sexes.

## Introduction

Pinniped life-history is often characterized by seasonal cycles of terrestrial (or ice bound) fasting (or reduced feeding) and at-sea foraging where energy reserves are replenished. Fasting periods coincide with periods of increased energy expenditure (whelping/breeding and moulting) and pinnipeds prepare for these periods by undertaking extensive foraging trips and feeding at depth [1]. Such activities prevent direct observation of feeding and consequently information on feeding success and prey consumption is difficult to obtain. A variety of methods have been developed to indicate important areas for feeding, for example the use of stomach or oesophageal temperature tags to record when the seal consumes prey (e.g., [2]–[5]), and video and image recording instruments to record prey encounters (e.g., [6]–[9]). Such devices can provide interesting information about foraging behaviour and intake rates, but often have limited sampling duration. A different method, offering long term (months) information of possible feeding success, is the investigation of pinniped diving behaviours by the use of satellite linked time-depth recorders (e.g., [10]–[18]). Indications of energy acquisition along the migration track can be observed through seasonal fluctuations in body composition, which should be reflected by changes buoyancy (e.g., [12], [18]–[24]). Seals do not have anatomical structures allowing them to regulate their buoyancy (below the depth where residual air in the lungs no longer has an effect [22]) and consequently, buoyancy is determined by the ratio of lipid to lean body tissue [21]. Blubber or lipid tissue is positively buoyant while lean tissue is negatively buoyant; hence an animal with a high ratio of lipid to lean tissue is more buoyant than an animal of similar mass but with a lower ratio of lipid tissue [18], [21]. Generally, seals store energy to be used during periods of fasting in an expanding layer of blubber, although they may also invest some of the energy attained during successful foraging by building core mass (i.e., muscles) (e.g., [25]). A particular dive profile, referred to as a “drift dive” has been identified in some pinniped species (e.g., northern elephant seals (*Mirounga angustirostris*) [10], [12], [19], [18], [23], southern elephant seals (*Mirounga leonina*) [20], [22], [26] and New Zealand fur seals (*Arctocephalus forsteri*) [27]). The shape of this dive type appears to be affected by changes in buoyancy, and therefore it can be used to provide information about where the seals are gaining relative lipid condition.

**Figure 1:**
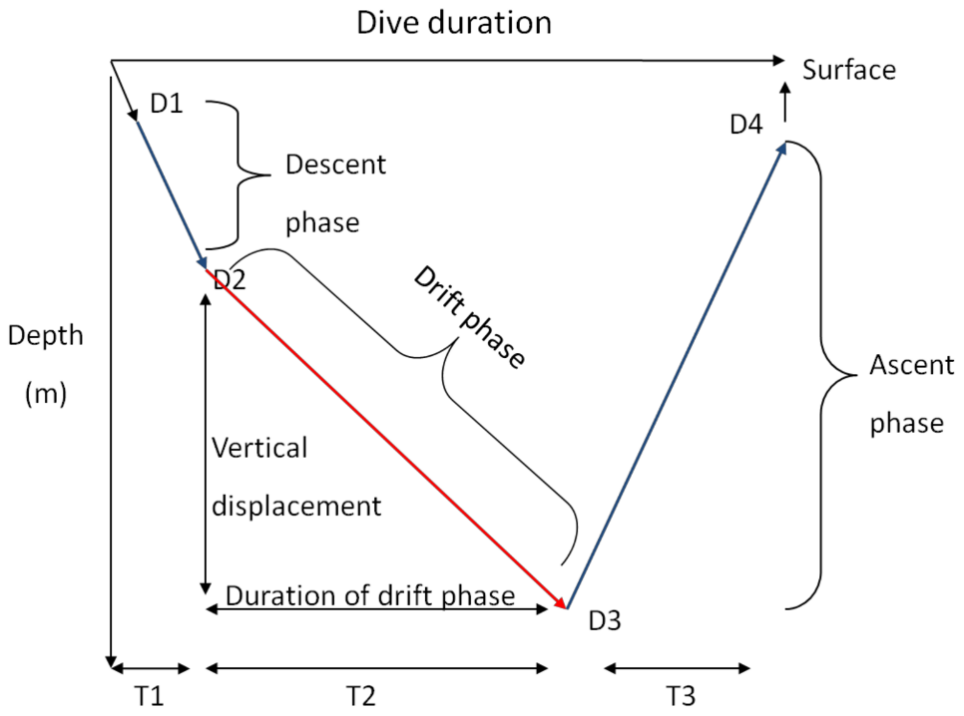
A description of a drift dive. Time spent from point D1 to D2 represents fast descent with possibly active swimming, D2 to D3 represents a dramatic decrease in vertical speed, with no active swimming and is called the “drift phase”. D3 to D4 represents ascent with active swimming. T1 and T3 represent the duration of ascent and descent and T2 is the duration of the drift phase. T1 to T3 is the total duration of the dive.

Drift dives are defined as a dive type with a direct descent to a depth at which point the descent rate decreases dramatically until the bottom of the dive, followed by a direct ascent to the surface ([10], [15], Fig. 1). In the case of very fat (blubber rich) seals; a “positive” drift dive may occur. This is when the seal is positively buoyant and drifts upward during the drift phase. However, this is quite seldom observed in adult animals, apart from elephant seal females in the late stages of pregnancy and juveniles at the beginning and end of their first foraging migration ([12], [22], respectively). Furthermore, a highly positive or negative buoyancy would increase energy expenditure during dives, and the optimal condition is probably at neutral buoyancy [28]. During drift dives the seals are thought to drift passively through the water column (no active swimming) [12], [18], [20], [21], [22], [23], and the dives have been hypothesised to represent periods of physiological processing such as digesting recently ingested food or for rest/sleep [10], [12], [15]. It has also been hypothesised that the form of this dive type, in addition to physiological functions, makes the seals less susceptible to predation, as the drift phase would start once they enter into the “safe zone” below possible predators' depth range (e.g., [23]).

Regardless of the function of these dives, the buoyancy will be determined by the difference between the density of the seal and the surrounding sea water [22]. Sea water density varies with salinity and temperature, and in less saline, ice covered waters this could result in a lower density of seawater causing the seal to have a higher vertical speed than in more saline waters. However, Biuw et al. [22] found that this only accounted for <1% of the overall bias when investigating drift diving in southern elephant seals. Drift rates will also be influenced by physiological and behavioural changes such as residual air in the lungs, drag and the orientation of the body in the water [22], [23]. Biuw et al. [22] found that residual air in the lungs accounted for the highest bias, and that this would depend on the depth where the drift segment would occur. They also found variability in drift rate at shallow dives (<100 m) suggesting that the seals voluntarily adjusted the volume of residual air in the lungs to optimise buoyancy during shallower dives. Furthermore, Mitani et al. [23] found that juvenile elephant seals would roll over on their backs and sink like a “falling leaf” during the drift phase, reducing the vertical speed to a minimum. These factors might influence the accuracy of identifying drift dives, and once the dives have been identified, the interpretation of the fluctuations in buoyancy can be challenging. A seal can invest acquired energy to improve their condition through somatic growth (lean tissue) or storing it as lipid tissue. It can therefore be difficult to interpret what a decrease in buoyancy represents. It could mean that a seal is foraging successfully and improving body condition by building core mass, or the seal could be experiencing poor foraging conditions, and as a result become skinnier (less blubber). However, despite these aspects of uncertainty, research has documented drift diving as an informative method to investigate the relative change in body composition over time for free ranging pinnipeds (e.g., [12], [18]–[20], [22]–[24], [29]).

**Figure 2:**
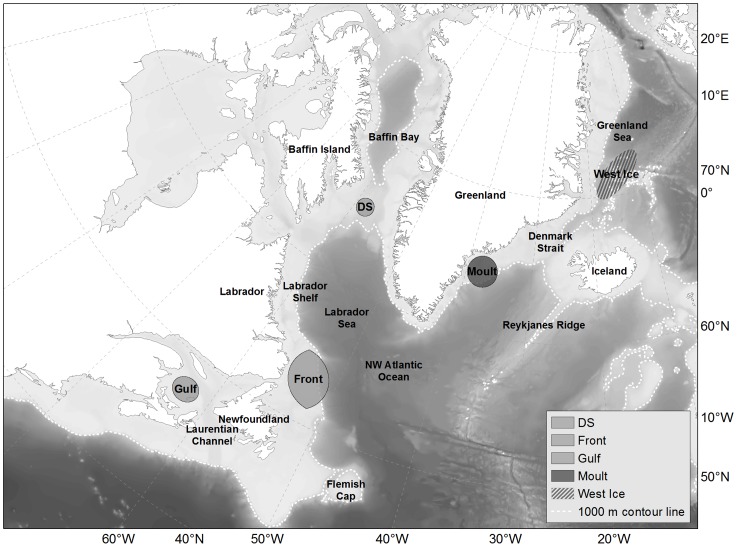
Map over the study area: Moulting area in southeast Greenland and breeding areas in Davis Strait (DS), The Front and The Gulf. Bathymetry of the study area is presented as backdrop in grey scale. Dashed white line is the 1,000

Hooded seals (*Cystophora cristata*) are sexually dimorphic, capital breeding pinnipeds distributed throughout much of the North Atlantic and adjacent Arctic Ocean [30]–[32]. They spend two months of the year fasting on the sea ice during breeding and moulting (March and July, respectively), and display a distinct annual migration pattern during the remaining 10 months (e.g., [14], [32]–[35]). North Atlantic hooded seals consist of two putative populations. Hooded seals whelping near Jan Mayen (the “West Ice”) are considered to constitute the Greenland Sea population while the Northwest (NW) Atlantic population, and the subject of this study, is comprised of animals whelping in Davis Strait, the Gulf of St. Lawrence (the Gulf) and at the Front (off Northeast Newfoundland and Southern Labrador) [30]. After the breeding period the NW Atlantic population leaves the whelping areas to feed before they eventually arrive at the ice off Southeast (SE) Greenland in June, just prior to the July moult [31]–[32], [34]–[35]. Post moult they disperse across the NW Atlantic and Baffin Bay before returning to the respective whelping areas ([33], Fig. 2).

The annual migration cycle for capital breeding pinnipeds is heavily influenced by the preparation for, or recovery from, whelping/breeding and moulting. Reproduction represents a period of increased energy demand (of variable length depending on species) and the cost of (or patterns of) mass gain and loss in relation to reproduction differ between males and females (e.g., [36]–[39]). Males must acquire more resources to attain, and maintain, their greater size [15], [40], especially when preparing for the breeding season and competition for females. Male hooded seals lose approximately 14% of their mean body mass (∼2.5 kg per day) over a breeding period lasting 2.5 weeks [41]. In comparison, females need to obtain sufficient energy stores to maintain pregnancy and prepare for a short and very intense lactation period. They wean their pup in only 3–5 days, during which time the mother loses on average 10 kg per day [42]–[43]. Thus, males and females lose a similar amount of energy during breeding, but females lose it in a much shorter time period than males, which can be expected to be more energetically demanding. The difference in energy expenditure over time and the change in total body composition could suggest that males and females have different foraging strategies in order to optimise their body conditions. Males may have greater demands for resources that can build core tissue, whereas females may have greater demands for resources that can be a rapid energy source for themselves and their pups. These possible differences in energy acquisition may be reflected by differences in their fluctuations of drift rates over time.

Previous analyses have shown that there is geographic segregation between high use areas by male and female NW Atlantic hooded seals [34], [44]). Andersen et al. [44] used First Passage Time (FPT, see [45]) and three habitat variables (geographic location, bottom depth and the Topographic Complexity Index (TCI) to identify such areas based on data from the same dataset as used in this study. FPT is defined as the time an animal uses to cross a circle of a given radius [45], of which the scale of the circle is determined by calculating the animals' average Area Restricted Search (ARS) scale (see [46]). Sex related segregation generally occurred during the post moult/pre breeding period in the northern areas of their range, where males had the longest FPT in the Davis Strait while females had their longest FPT along the Labrador shelf and over the Labrador basin [44]. All seals had a long FPT in SE Greenland, near the moulting area [44]. The Gulf breeding animals exhibited a high degree of overlap between males and females in the Gulf of St. Lawrence during the post breeding period, but they mixed with the rest of the population in SE Greenland in time for the moult [44].

The objective of this study was to determine if hooded seals exhibit drift dives and if so, to use this information to extract information about how these animals' buoyancy changes over time. A difference in drift diving frequencies across months may be a reflection of different foraging strategies carried out by males and females. We can thereby learn about how males and females allocate energy resources differently to building relative lipid body mass in relation to important life history events such as whelping/breeding and moulting. A change in drift rate may further identify specific locations where they experience an increase in relative lipid tissue, i.e., foraging success. Also, if the FPT method correctly identify important seal foraging areas, we expect to observe a positive change in drift rate in areas with longer FPTs (i.e., more time spent in a limited area).

## Methods

### Ethics statement

The capture and tagging protocols were reviewed and approved by the Canadian Council of Animal Care. Capture and deployment of satellite transmitters on seals were carried out under appropriate animal care permits and by experienced personnel with the Department of Fisheries and Oceans (DFO), Canada. This project did not have any adverse effects on the environment. The permit numbers were NAFC 2004-11 and NAFC 2008-04.

### Deployment of Satellite Relay Data Loggers (SRDLs)

The study area was the NW Atlantic Ocean, extending from the Gulf of St. Lawrence northwards covering most of Baffin Bay, including Davis Strait, and along the Greenland shelf to SE Greenland (Fig. 2). Satellite transmitters were deployed directly after moulting in SE Greenland during three field seasons (2004, 2005 and 2007; 65°N, 37°W), and after breeding/whelping during three field seasons (The Front: 2004 and 2008; 49°N, 52°W; The Gulf: 2004, 2005 and 2008; 46°50′N, 62°W). In total, 51 adult seals were tagged of which there were 33 adult females and 18 adult males. All hooded seals were captured using a V-shaped pole-net on the ice. They were weighed, and subsequently tranquilized using a single injection of tiletamine hydrochloride and zolazepam hydrochloride (Telazol, AH. Robins Company, Richmond, VZ, USA) administered intramuscularly (1 mg·kg_(body mass)_^−1^). Satellite Relay Data Loggers (SRDLs; Sea Mammal Research Unit (SMRU), St. Andrews, Scotland) were glued to the head or upper neck of the seal, using quick drying epoxy glue (Cure 5, Industrial Formulators of Canada Ltd. Burnaby, BC Canada). The size of the SRDL is 10.5×4 cm, weighing approximately 370 grams. The procedure was completed within 20 minutes and the seals were monitored until they recovered from the tranquilizer.

The SRDLs collect a range of behavioural information about free ranging animals at sea. The data included number of dives, dive depth (deeper than 6 meters), dive duration and surface intervals, along with detailed information of the time-depth profile for each dive. Time and depth were recorded every 4 seconds throughout the duration of the dive and these full resolution profiles were compressed on-board using a broken-stick abstraction algorithm, resulting in four at-depth points plus two surface points (start and end) [13], [47]. Finally, data were compressed before transmission via the ARGOS satellite system ([13], [47]–[48]. Transmissions were attempted every 80 seconds when the seals were at the surface. The data was filtered prior to analysis to remove outliers by using an algorithm based on the travelling speed of the tracked animal, distance between successive locations and turning angles [49]. We used a maximum swim speed of 2 m/s between successive locations which was similar to that used for grey seals [50].

### Diving and drift dives

Drift dives were identified by extracting vertical speeds from all time-depth profile segments having characteristics of drift dive segments [51]. The depth resolution for the full resolution dive profile was 0.1 m. Dive profiles were reduced to four inflection points excluding surface points (e.g., [47]). Two vertical speeds from the segments before and after the deepest point of the dive were extracted. A segment was classified as a possible drift segment when the maximum dive depth was deeper than 50 m, absolute vertical speed of the dive segment was between 0.6 and 0 m/s, the length of the segment was longer than 3 minutes and when the segment constituted more than 40% of the total dive duration (Fig. 1). Only descending segments (before the deepest point of the dive) fulfilled these criteria. A selection of dives, which included such potential drift dive segments, were also investigated visually, so that we could be certain that our selection would be representative of drift dives and reduce the likelihood of bias in selected dives.

The chosen inflection points do not represent true start or end points of linear time-depth segments; instead they rather represent points at which the dive profile changed its shape most significantly [52]. Consequently, the true start and end of a drift phase are not known, and the extracted vertical speed from the inflection points will have an error component. We therefore used a robust iterative smoothing method to remove outliers in our time series of extracted vertical speeds (e.g., [53]) assuming that the drift rate changes slowly over time and not in sudden jumps. This method calculated a weighted running mean with a Gaussian kernel of sigma = 4 days for each time step. Vertical speeds deviating more than 0.15 m/s from the smoothed values were rejected from the time series and the process was repeated. After three to four steps no more outliers were found and the remaining vertical speeds were used to calculate a weighted running mean, which we assume to be representative of drift rates. These drift rates were then used to extrapolate daily drift rates and daily changes onto the complete time series. Every dive therefore had an associated drift rate and dives with a dive segment fulfilling the above criteria, as well as an associated vertical speed within 0.06 m/s of the daily drift rate value, were classified as potential drift dives.

The percent frequency (i.e., number per unit time) of drift dives throughout the 24 hour period and by month was investigated. The objective was to identify the timing and frequency of drift diving. The sunlight hours vary significantly at Arctic latitudes, and may affect diving behaviour more than the actual time of day. We therefore calculated the sun angle at the location of dive occurrence to investigate how sunlight affected dive behaviour on a diurnal basis. Random intercept models were used to investigate regular diving behaviour (dives with no drift component; maximum dive depth and dive durations) on a diurnal scale and drift diving frequencies on a diurnal and monthly scale. Individual seal id was implemented as a random effect in the model, but with the assumption that the variation around the intercept was normally distributed within a certain variance for each individual seal [54]. The models were fitted using the lme function in the nlme library in R (http://www.R-project.org).

A GAM was fitted to generate spatial predictions of the daily change in drift rates throughout the annual migration. The models were run on two datasets (aggregated dataset based on ARS (FPT dataset), and the full drift dive dataset). The effects of FPT, geographic location and day of year were used as predictor variables, and the daily change in drift rate was set as the response variable. The FPT dataset was averaged across an 80 km step (based on a 40 km ARS scale used in Andersen et al. [44]) and FPT was calculated per step yielding 1,459 data locations. The GAM was implemented with the gam function in the mgvc library in R [55]–[56] using restricted maximum likelihood (REML) as the fitting method. Random effects were implemented by using the “re” smoother option, which is appropriate for simple, independent random effects [57]. We were therefore running a GAM including random effects (individual seal id) instead of a mixed GAM (GAMM). Sexual segregation in the data was investigated by including sex as an interaction term.

To select between competing models we applied an information-theoretic approach and examined parameter weightings using Akaike's Information Criterion (AIC). All models (14) nested within the full model were assumed to be candidate models, and models with Δ_i_<2 are considered to have substantial support while Δ_i_>10 have very little support (Δ_i_ is the difference between the AIC of the best fitting model and that of model *i*; [58]). If the addition of one predictor variable to a model resulted in an AIC of <2 values from the model without this variable, and the model fit was not improved (deviance explained), the added variable was deemed a pretending variable and removed from the analysis [59].

All maps were created using ArcGIS Desktop 10 (Environmental Systems research Institute, Redlands, CA) and the bathymetric backdrop and 1000 m depth contour line was derived using bathymetry data from the General Bathymetry Chart of the Ocean (GEBCO; http://www.gebco.net/).

## Results

In total, we examined 87,565 dives from 51 adult seals with complete dive records. Of these, 6,806 dives from 47 seals fitted our selection of criteria for drift dives (30 females and 17 males; Table S1). The number of drift dives extracted per seal ranged from 16 to 846 (Mean = 144.81±156.97) depending on tag survival time and individual variability in drift diving occurrence. The mean maximum drift dive depth across all seals was 199.25±95.10 m and the mean drift dive duration was 15.03±5.38 min.

### Diurnal dive behaviour

**Figure 3:**
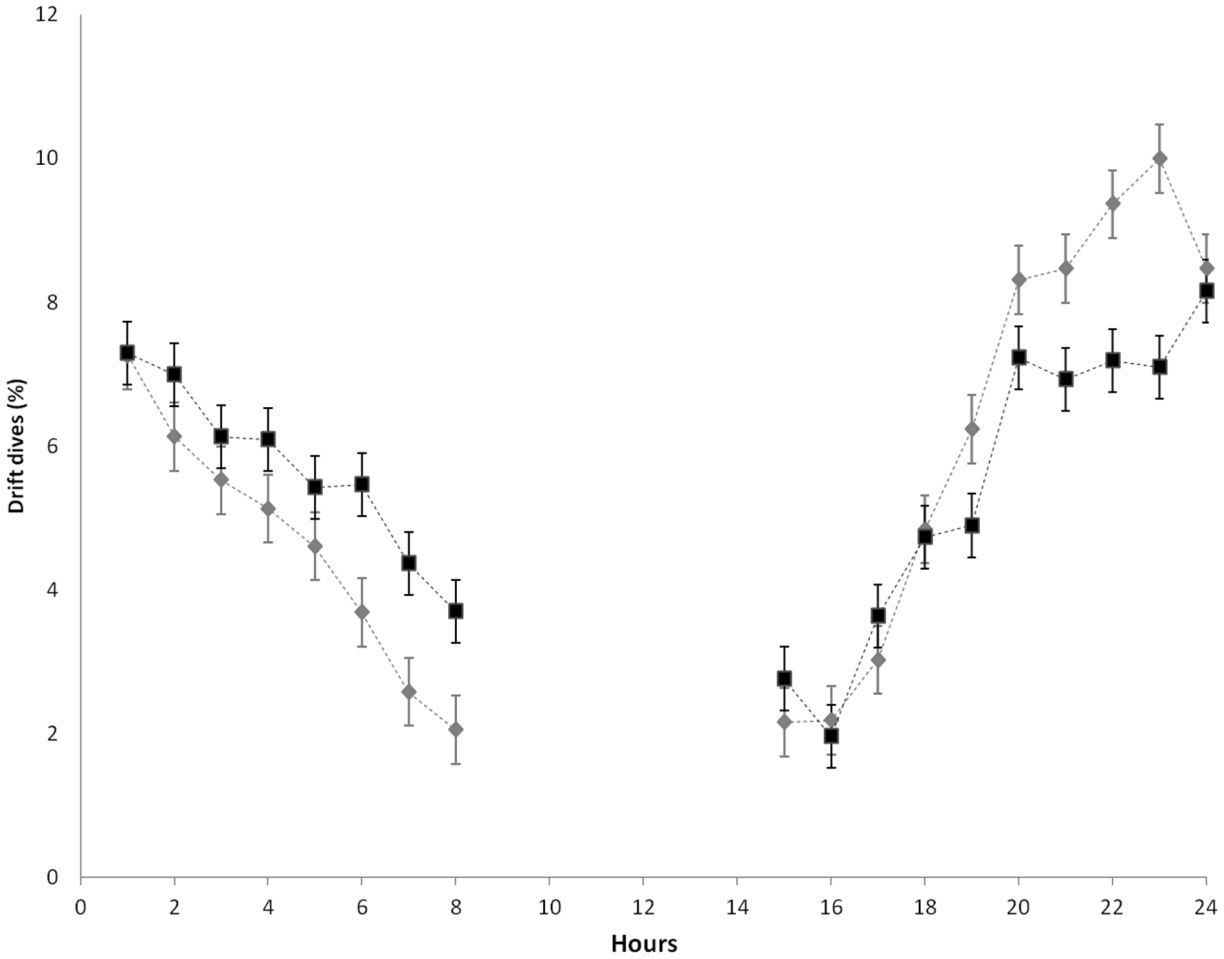
Percentage of drift dives as a function of time of day (1–24 = 01:00–24:00) across females (grey; n = 30), and males (black; n = 17). There were no drift dives observed between 08:00 and 15:00. Error bars represent the standard error.

The occurrence of regular dives (i.e., dives with no drift component) did not change in accordance with the sun's angle to dive locations (Fig. S1). In contrast, there was a clear diurnal pattern observed for drift dives where 80% of the dives occurred when the sun was below the horizon (Fig. S1) and no drift dives were observed during the day between 08:00 and 15:00 (Fig. 3).

Drift diving occurrence by hooded seals varied significantly throughout the 24 hour cycle (F = 6.59, p<0.0001, df = 17), and a significant level of sexual segregation was found (F = 1.71, p<0.05, df = 17), where males performed more drift dives than females between 01:00 and 08:00 and females performed more drift dives than males between 19:00 and 23:00 (Fig. 3).

Dive duration and maximum dive depth of regular dives were highly correlated (r = 0.74, p<0.001) and both sexes dived significantly deeper and longer during mid day (F = 66.62, p<0.001, df = 23; F = 178.61, p<0.001, df = 23, respectively, Fig. S2 a, b).

### Drift diving behaviour by month

**Figure 4:**
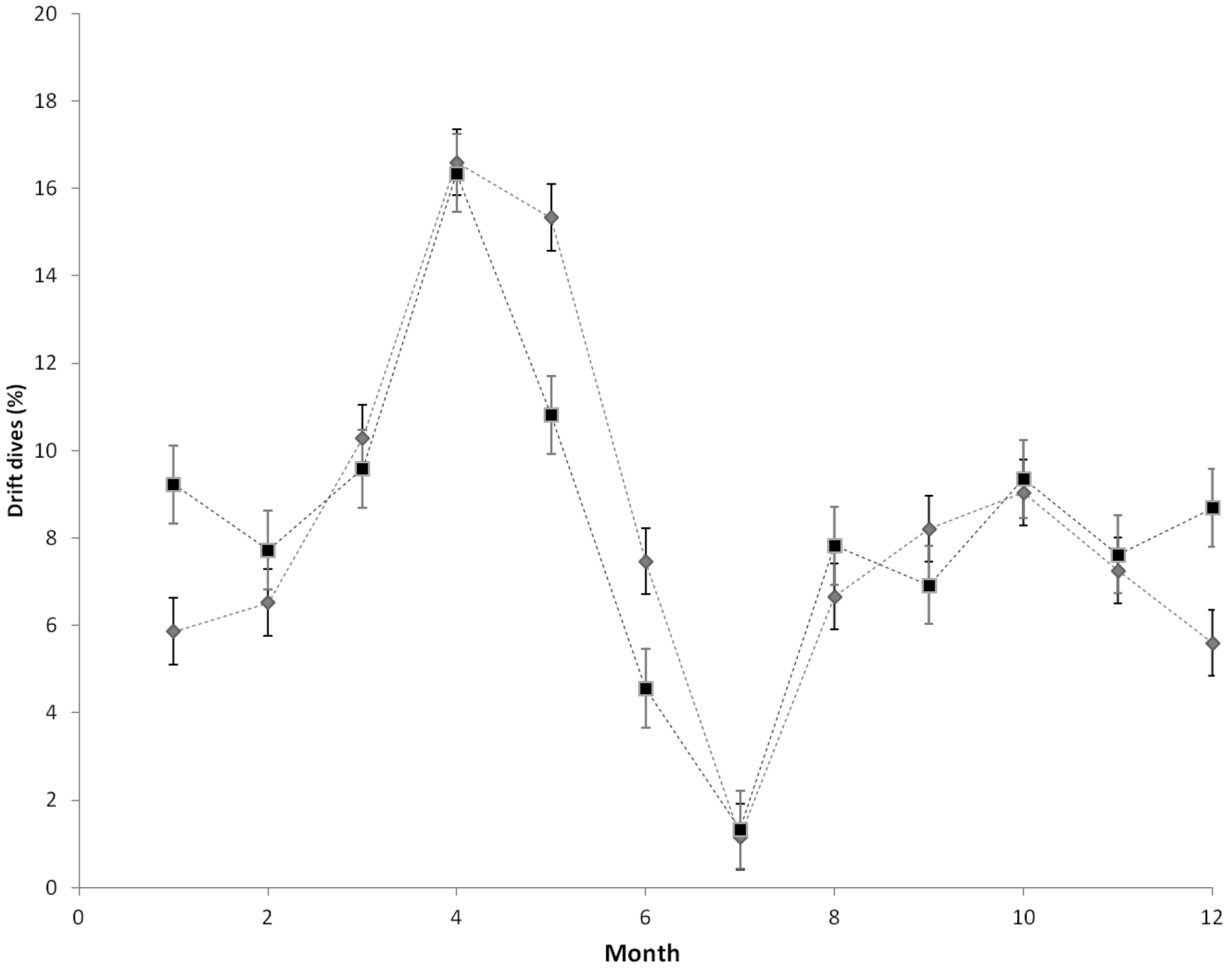
Percentage of drift dives as a function of month (1–12 = January–December) across females (grey; n = 30), and males (black; n = 17). Error bars represent the standard error.

Using a random intercept model we detected a significant difference in drift dive frequencies between months (F = 10.93, p<0.0001, df = 11). Males and females carried out a similar amount of drift dives per month and there was a clear increase in drift dive occurrence after breeding in April and May, for both sexes (Fig. 4).

### Daily change in drift rate

**Figure 5:**
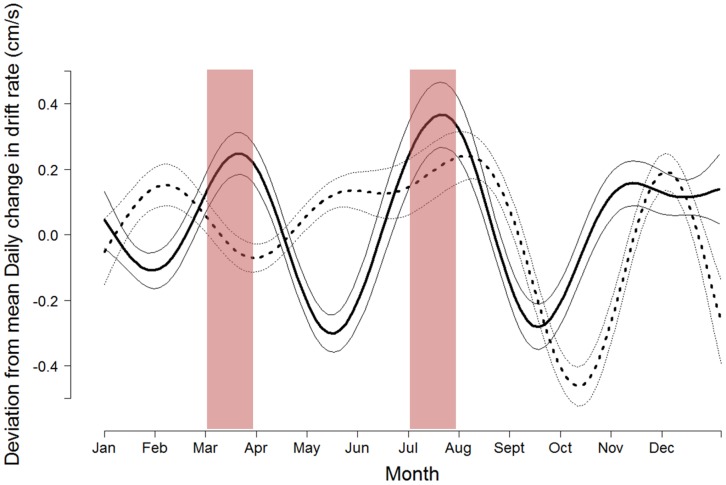
Predicted GAM results of the deviation from the mean daily change in drift rate (cm/s) over time, where the mean is at 0. Data from males and females were here run in seperate models: males: solid black line (n = 17) and females: dashed line (n = 30). Thin lines represent the standard error and red columns represent the annual fasting periods (breeding/whelping in March and moulting in July).

Of a total of 6,806 drift dive locations, the averaged FPT data, aggregated at the ARS scale of 40 km, yielded 1,459 data points. We ran 14 candidate models on each of the datasets, identifying FPT as a pretending variable. We therefore disregarded the aggregated dataset and focussed this analysis on the full drift dive dataset of 6,806 dive locations. The best GAM model indicated that seasonality (represented by the day of year) and the geographic location of the seals best explained the variation in the daily change of drift rates (AIC*w_i_* = 1, deviance explained = 29.7%; Table S2, Fig. S3). All other models were highly implausible (ΔAIC>10). To further investigate the segregation between males and females identified in the best model, we decided to run the GAM model on males and females separately and found that the model investigating the daily change in drift rate by geographic location and day of year was a better fit for males than for females (deviance explained = 35.1% and 19% respectively, Fig. 5). A difference in the timing of relative lipid loss or gain (as represented by changes in drift rates) was observed, although, both males and females experienced a large negative change in drift rate (i.e., reduced buoyancy) directly after the moult (Fig. 5). Females experienced a loss of buoyancy in December, but regained lost buoyancy by February. They then exhibited some loss in relative lipid condition directly prior to the breeding period. Males showed level buoyancy through the winter, but a decrease occurred in January before a continued increase in the relative lipid condition was observed up to the start of the breeding period. They further experienced a large negative change in buoyancy directly after the breeding period was over, while females started to increase their relative lipid condition at an earlier stage than males. Both males and females experienced a gain in relative lipid condition at the end of June and beginning of July, immediately before the moult (Fig. 5).

**Figure 6:**
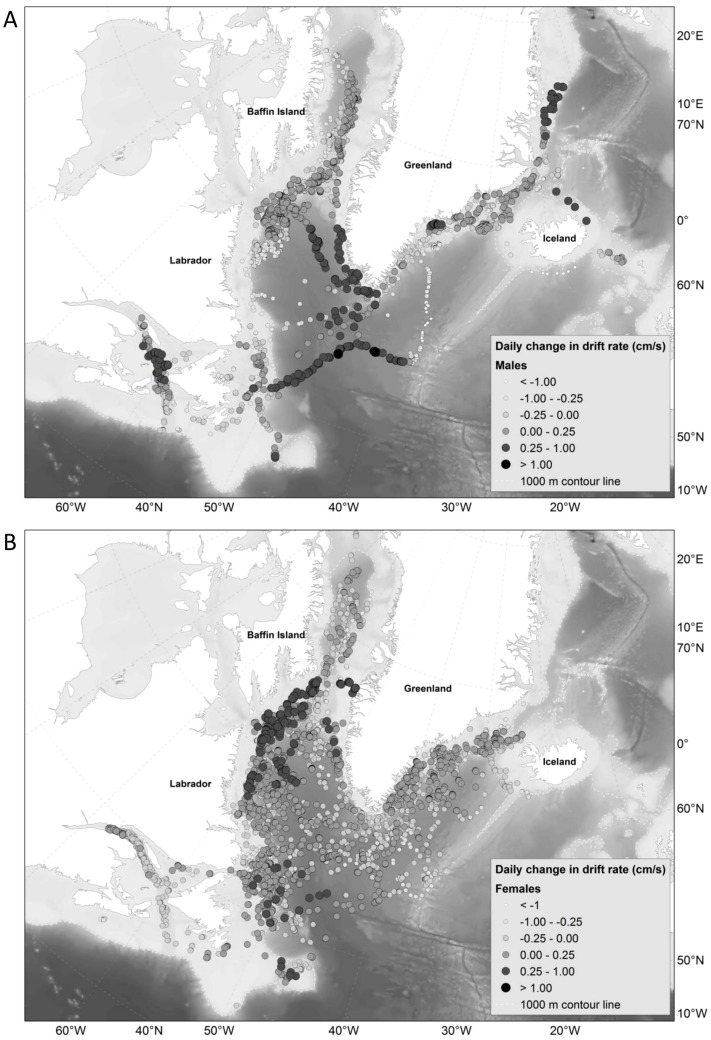
Plot of fitted values (daily change in drift rate (cm/s)) from the best GAM for a) males (n = 17) and b) females (n = 30). The darker the colour, the larger the daily change in drift rate.

We mapped the fitted values from our best GAM to identify and compare the geographic locations of increased relative lipid content as shown by the change in buoyancy for males and females (Fig. 5). Males experienced a positive change in drift rate (i.e., increased buoyancy) throughout their range (daily change 0–0.25 cm/s), although, 11 individuals in the southern part of the range experienced a higher positive change (the Gulf, southern Labrador Sea, Southwest and East Greenland (daily change of >0.25 cm/s), Fig. 6a). Females seemed to experience a large positive change in buoyancy in the northern parts of the Labrador shelf and in the Davis Strait, but also around the Front and Flemish Cap area (daily change >0.25 cm/s). However, positive change occurred on and off the Labrador shelf as well as in SE Greenland. Females exhibited a lower level of positive change in the Gulf than males (daily change <0.25 cm/s, Fig. 6b).

## Discussion

Drift diving has been identified in a number of other pinniped species and this study shows that hooded seals of the NW Atlantic Ocean also carry out this specific type of dive. Hooded seals exhibited a clear diurnal pattern in dive duration and depth of dives, where regular dives were longer and deeper during the day than at night (Fig. S2 a, b), although the frequency of dives were similar between night and day (Fig. S1). These findings are similar to what Folkow and Blix [60] found for the Greenland Sea population and they suggested that hooded seals are foraging on diurnally migrating prey that approach the surface at night and migrate down during the day. Drift dives occurred with highest frequency during the time of day when the seals exhibited the lowest dive duration and shallowest dives (i.e., night time; Fig. 3). Crocker et al. [12] suggested that northern elephant seals would drift while processing food, thereby balancing the oxygen demands of foraging and locomotion while diving continuously and remaining within their aerobic limits. Seals have further been found to be able to delay costly physiological processes (e.g., digestion) that are incompatible with the physiological adjustments to diving until hours after periods of active foraging [61]. It is not possible for us to infer the function of drift dives for hooded seals based on diurnal diving behaviour without further analysis. Crocker et al. [12] hypothesised that drift diving is a behavioural response to increased feeding success, however, the seals' ability to invest attained energy to somatic growth (resulting in decreasing buoyancy), makes the relationship between increased drift dive frequencies and foraging difficult to interpret with certainty.

Data on the exact timing and duration of the moult are scarce [32] but we found that the time period where no tags were active (lost as a result of the moult, or not attached because the moult was not yet finished), occurred between June 27^th^–July 20^th^, which suggests that the moult started at the end of June for some seals and ended towards the last week of July for others. After the moult, both sexes showed a large decrease in buoyancy, indicating increasingly poor body condition. Rasmussen [32] reported that the total reduction in blubber mass during moulting would vary depending on the condition of the seals at arrival to the moulting grounds. Female hooded seals seemed to start to store lipid energy directly after breeding, steadily increasing their buoyancy towards the moult. Males showed an initial decrease in buoyancy, which may indicate that they use the first half of this same period to allocate ingested resources preferentially towards somatic growth, (i.e., relative lean body mass). According to our results males did not start to increase their relative lipid condition until late May (Fig. 5). These findings are supported by Thordarson et al. [62] who reported that males were heaviest in core mass (i.e., muscle) in May. They further found that core mass decreased by 14% between May and August and that the males were 10% heavier in October than August. This weight gain was a result of increased relative lipid content and the increased buoyancy was reflected in our model results (Fig. 5). Males exhibited a negative change in buoyancy post moult, followed by increased buoyancy from mid September to November (Fig. 5).

The duration of the moult is similar for both males and females, as opposed to breeding when females invest on average 4 days [42] and males' ∼2.5 weeks [41]. This suggests that, although the energy lost during fasting periods is similar between sexes, the timing of loss may be different during breeding, but similar during the moult. Males may also be freer to enter the water and occasionally feed during breeding, as opposed to the moulting period when both sexes fast for a similar amount of time. It has been suggested that the energetic costs associated with the immediate migration after breeding followed by reduced food intake during the moult must be met from energy stored prior to the breeding season or from what have been acquired during the migration between breeding and moulting [63]. The relatively poor body condition after the moult (or less relative lipid content) observed for both sexes suggests that the post moult migration marks the beginning of the build up of energy reserves to prepare them for breeding and consequently the moult.

Among sexually dimorphic pinnipeds, males store energy to last through the period of reduced intake during the breeding period and to improve their ability to compete for mates (e.g., [38]). Due to the larger size of males, it is expected that they need to feed more than females if they are foraging on similar prey [15]. Although the drift dive frequencies were similar directly after breeding, males have previously been found to perform slightly longer and deeper dives than females during the same time period (see [44]). Our habitat model also showed that male and female NW Atlantic hooded seals have different movement patterns, using different geographical areas. These findings could indicate that males and females avoid competition by foraging on similar prey at different depths where males seek out larger prey of the same species found deeper in the water column, or that they feed on different prey. Tucker et al. [64] found evidence of variation in diets, diet quality and breadth, reflecting different foraging strategies by males and females during the pre and post breeding periods. However, the most important prey item for both sexes post breeding were redfish.

After the moult, males appeared to recover relative lipid stores faster than females by exhibiting an increase in buoyancy from mid September, while females did not experience an increase in buoyancy until a month later (mid October, Fig. 5). Andersen et al. [44] found that males undertook longer dives during this period, but the dive depths were similar between sexes throughout the winter months. This further suggests that hooded seals exhibit a resource partitioning strategy through spatial segregation. Our best model results (geographic location and day of year influencing the fluctuations of buoyancy) also showed a higher deviance explained for males, supporting earlier findings that males seem to be more spatially localised in their habitat use than females [34], [44].

Females experienced two periods of negative change in buoyancy during the post moult/pre breeding period (December and February, Fig. 5). The buoyancy at these times may have been negatively influenced by the increasing lean weight of the growing foetus (e.g., [18], [24], [40]). As the foetus is growing, the female would need to counteract the added relative lean mass by increasing her relative lipid condition to maintain neutral buoyancy, which may explain the decreased drift rate observed in December, followed by an increase in January and another decrease in February (Fig. 5). Robinson et al. [24] reported a marked decline in the daily change in drift rates well before the return phase of the migration in female elephant seals. Although hooded seal pups have been found to be born with an average of 15.9±3.10% fat [63] which is higher than what has been found for elephant seals (∼4%, [65]), the negative influence on the female hooded seals' buoyancy may still be detectable. We would also expect females to show an initial negative change in drift rate post breeding due to the buoyancy lost during lactation (females have been found to lose on average 10 kg per day during the approximate 4 days of lactation; [43]). Thus the rapid loss of relative lipid content by females vs. the more gradual loss by males (2.5 weeks, 2.5 kg per day; [41] during breeding may trigger behavioural differences in feeding strategies which could be reflected in the dive behaviour.

In this study we explored the relationship between areas where seals spend more time (i.e., longer FPT) (see [34], [44]) presumably to feed, and areas where seals appeared to be successfully feeding, assuming that resources are allocated preferentially to lipid storage (which we know are not always the case). Although we observed some area overlap, FPT did not explain changes in hooded seals' buoyancy during migration. Hooded seals generally started their post fasting migration by transit to locations where they slowed down their passage time, which was reflected in an initial decrease in body condition (negative change in daily drift rate) (Fig. 5; Fig. S4, S5, S6, S7). Elephant seals also show periods of decrease in body condition during migration, which has been linked to long travelling distances and short travel time [20], [22]. However, reasons for why FPT did not explain changes in buoyancy could be due to hooded seals' ability to forage successfully regardless of passage time (observed increased buoyancy). In addition, an observed decrease in buoyancy does not necessarily mean that the seal is experiencing poor foraging conditions, but that they invest energy intake to building core mass. Seals have also been found to return to areas of successful foraging based on previous experience, where they slow down and increase their turning rate without, necessarily, an increase in prey encounters [17].

In summary, this study has shown that NW Atlantic hooded seals exhibit drift dives, as also observed in a number of other pinnipeds. Although it is difficult to directly observe hooded seals and how they interact with their environment, examining these dives have provided valuable information on where, and when, hooded seals experience seasonal changes in relative lipid content and allowed us to relate these changes to important life history events such as whelping/breeding and moulting. Differences in seasonal fluctuations of buoyancy between males and females suggest that they respond differently to the annual cycle of fasting periods and energy acquisition.

## Supporting Information

Figure S1 **Occurence of regular dives (i.e., dives with no drift component) to the left (n = 87,565) and drift dives to the right (n = 6,806) in realtion to the suns angle to the dive location throughout the day.** The boxes represent the interquartile range and the solid dark line is the median. Whiskers are 1.5 times the interquartile range and outliers are represented by open circles.
(TIF)

Figure S2 a) Dive durations (seconds) during regular dives as a function of time of day (01:00–24:00) across females (grey lines), and males (black lines). b) Mean maximum dive depths (meters) during regular dives as a function of time of day (01:00–24:00) across females (grey lines), and males (black lines). Females: n = 30, males: n = 17. Error bars represent the standard error.
(TIF)

Figure S3 **Predicted GAM results of the deviation from the mean daily change in drift rate (cm/s) over time, where the mean is at 0.** Males and females are here run in the same model: males: solid black line (n = 17), females: dashed line (n = 30). Thin lines represent the standard error and red columns represent the annual fasting periods (breeding/whelping in March and moulting in July).
(TIF)

Figure S4 **All individual females tagged in July (n = 11).** Left hand panels show the fluctuation in drift rate (m/s), fitted with a smooth line. Blue lines represent 1 standard error from the smooth. The right hand panel shows the daily change in drift rate (cm/s) over the same period. The title of each plot is the individual seal id.
(TIF)

Figure S5 **All individual males tagged in July (n = 7).** Left hand panels show the fluctuation in drift rate (m/s), fitted with a smooth line. Blue lines represent 1 standard error from the smooth. The right hand panel show the daily change in drift rate (cm/s) over the same period. The title of each plot is the individual seal id.
(TIF)

Figure S6 **All individual females tagged in March (n = 20).** Left hand panels show the fluctuation in drift rate (m/s), fitted with a smooth line. Blue lines represent 1 standard error from the smooth. The right hand panels show the daily change in drift rate (cm/s) over the same period. The title of each plot is the individual seal id.
(TIF)

Figure S7 **All individual males tagged in March (n = 9).** Left hand panels show the fluctuation in drift rate (m/s), fitted with a smooth line. Blue lines represent 1 standard error from the smooth. The right hand panels show the daily change in drift rate (cm/s) over the same period. The title of each plot is the individual seal id.
(TIF)

Table S1 **Tagging information for the 47 seals.** ID = individual tag numbers, Sex = males (M) or females (F), Wt = weight in kg at the time of tagging, Start = tag date, End = date when tag stopped transmitting, Days = number of days of transmissions, Latitude and Longitude = tag location.
(DOC)

Table S2 **AIC table.** The response variable, daily change in drift rate, was investigated in relation to geographic location and day of year. Loglik is the loglikelihood, K is the number of parameters in the model. AIC_i_ is AIC for model *i*, and ΔAIC is the difference between the AIC of the best fitting model and that of model *i*. Exp(−0.5Δ_i_) represent the relative likelihoods and the *w*_i_ is the Akiake weights. D.E% is the deviance explained by the model. Models with and without sex as an interaction term was run.
(DOC)
