# Supplementary figures and images for: Drift Diving by Hooded Seals (Cystophora cristata) in the Northwest Atlantic Ocean
Source: PLoS One. 2014 Jul 22;9(7):e103072. doi: 10.1371/journal.pone.0103072 (PMC4106908; doi:10.1371/journal.pone.0103072)

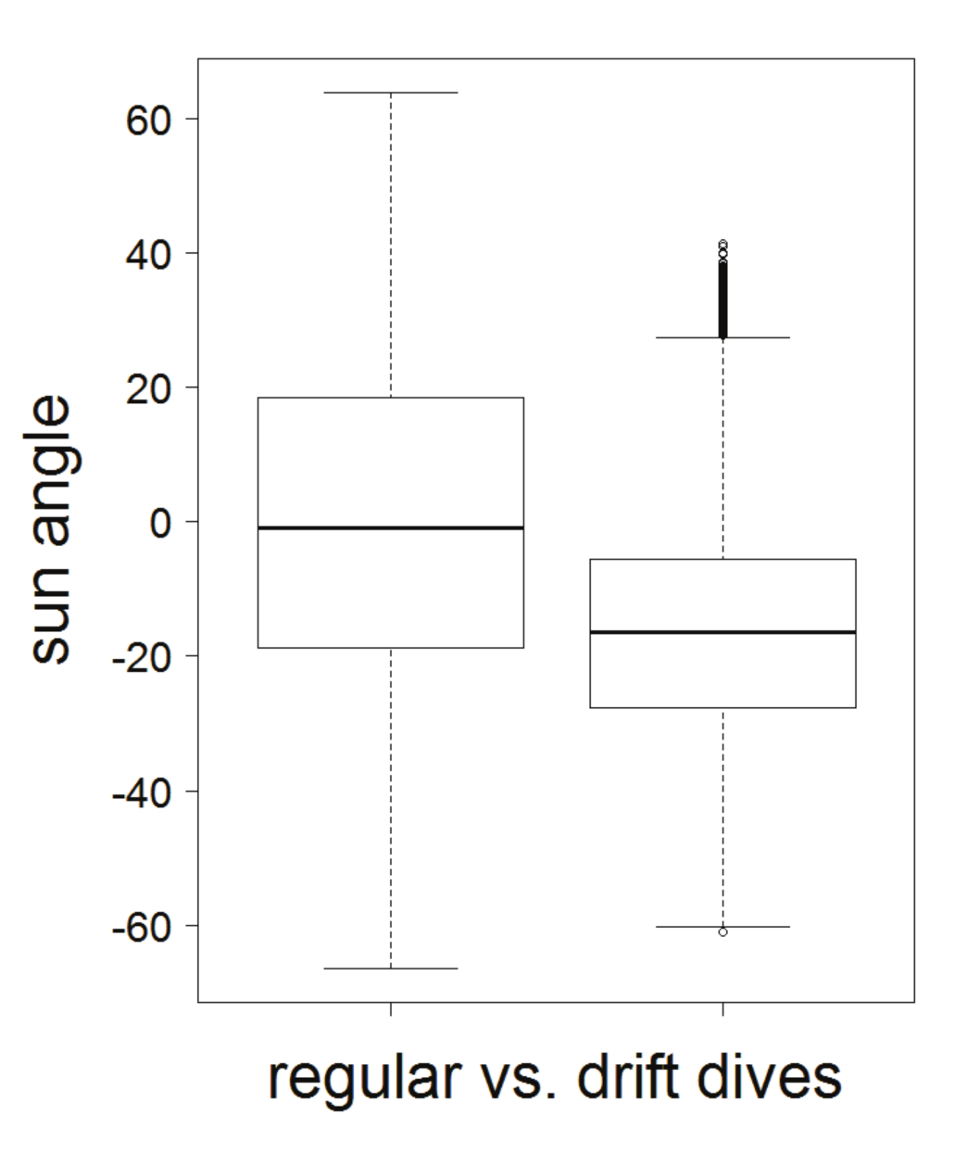

Supplement: Figure S1 — Occurence of regular dives (i.e., dives with no drift component) to the left (n = 87,565) and drift dives to the right (n = 6,806) in realtion to the suns angle to the dive location throughout the day. The boxes represent the interquartile range and the solid dark line is the median. Whiskers are 1.5 times the interquartile range and outliers are represented by open circles. (TIF) [file pone.0103072.s001.tif]

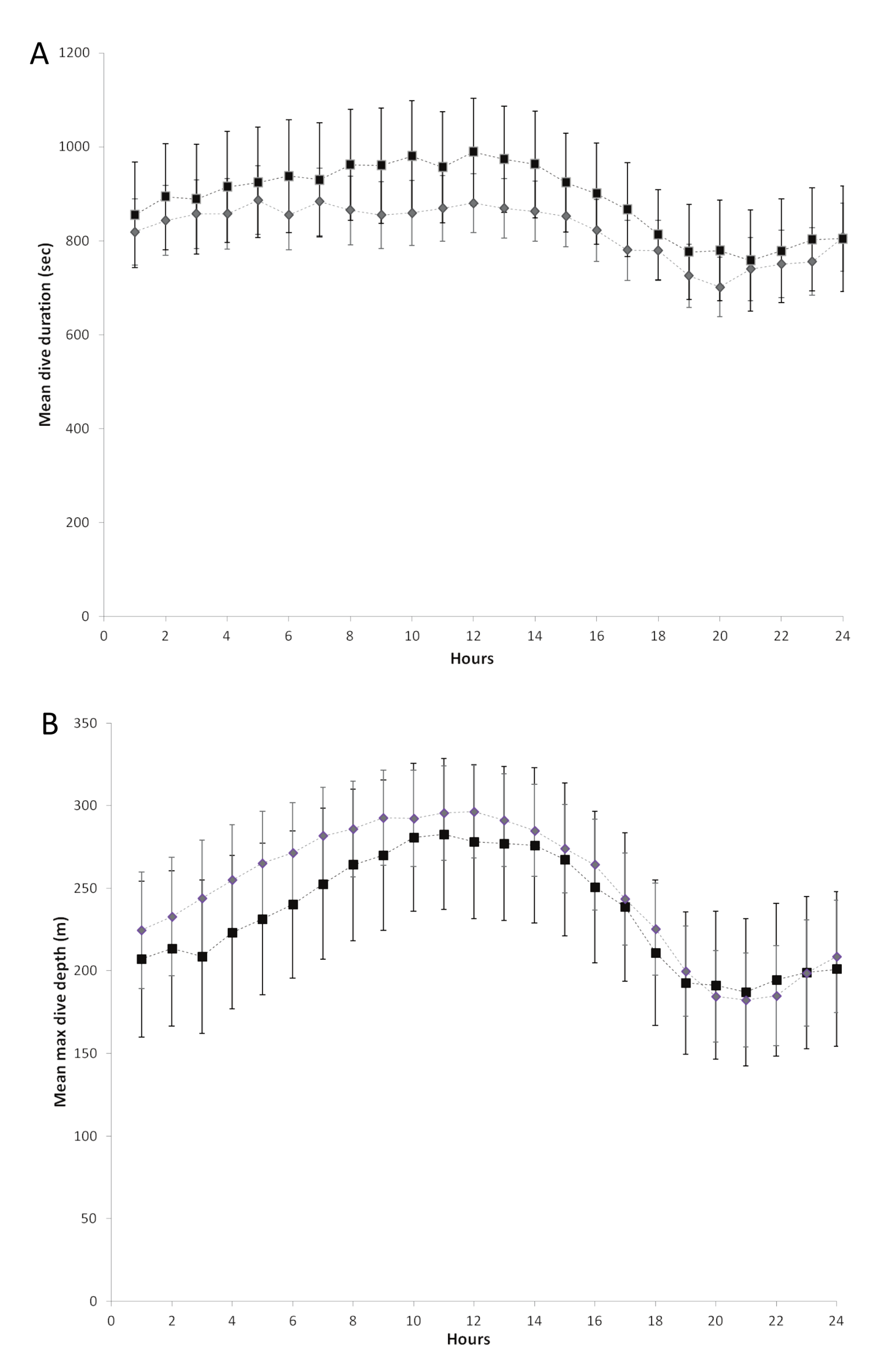

Supplement: Figure S2 — a) Dive durations (seconds) during regular dives as a function of time of day (01:00–24:00) across females (grey lines), and males (black lines). b) Mean maximum dive depths (meters) during regular dives as a function of time of day (01:00–24:00) across females (grey lines), and males (black lines). Females: n = 30, males: n = 17. Error bars represent the standard error. (TIF) [file pone.0103072.s002.tif]

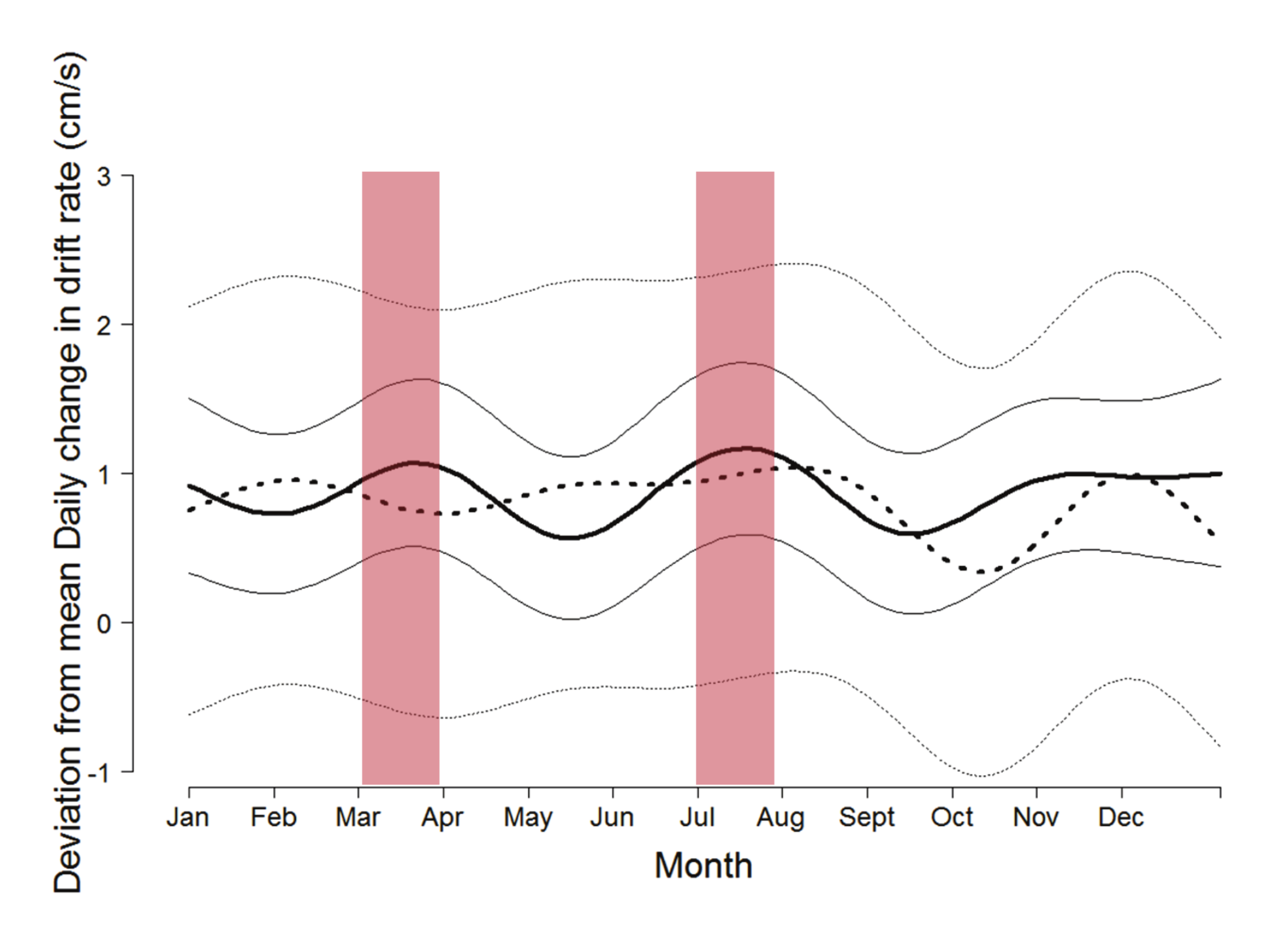

Supplement: Figure S3 — Predicted GAM results of the deviation from the mean daily change in drift rate (cm/s) over time, where the mean is at 0. Males and females are here run in the same model: males: solid black line (n = 17), females: dashed line (n = 30). Thin lines represent the standard error and red columns represent the annual fasting periods (breeding/whelping in March and moulting in July). (TIF) [file pone.0103072.s003.tif]

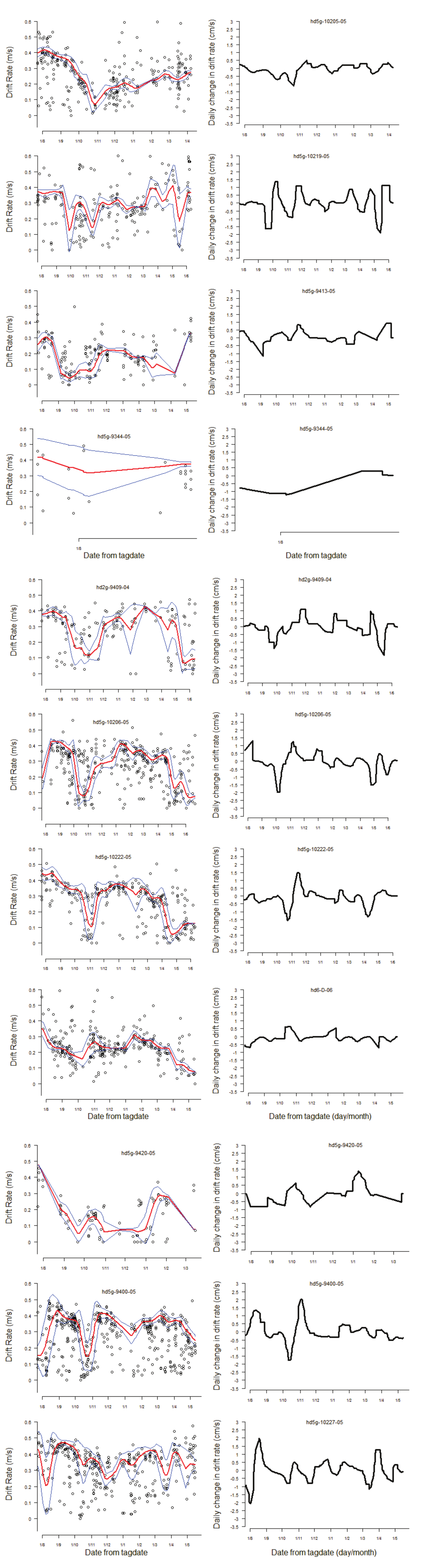

Supplement: Figure S4 — All individual females tagged in July (n = 11). Left hand panels show the fluctuation in drift rate (m/s), fitted with a smooth line. Blue lines represent 1 standard error from the smooth. The right hand panel shows the daily change in drift rate (cm/s) over the same period. The title of each plot is the individual seal id. (TIF) [file pone.0103072.s004.tif]

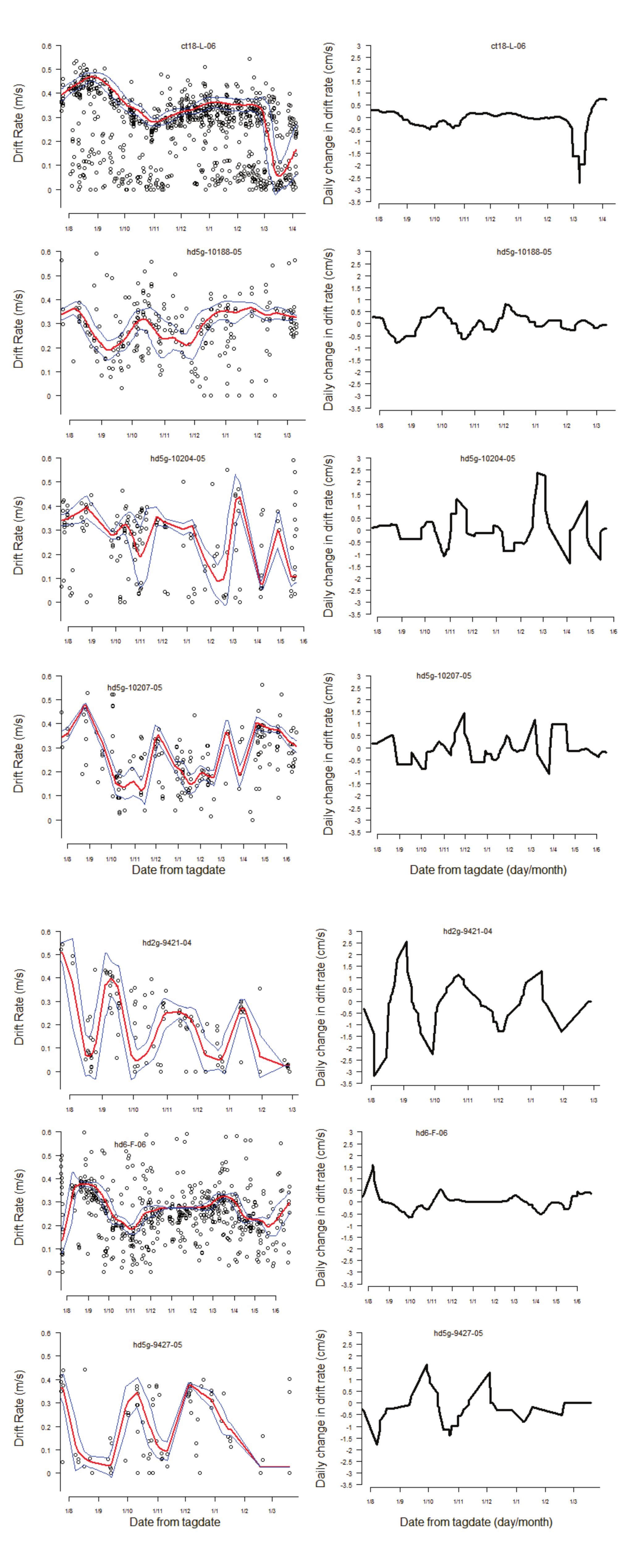

Supplement: Figure S5 — All individual males tagged in July (n = 7). Left hand panels show the fluctuation in drift rate (m/s), fitted with a smooth line. Blue lines represent 1 standard error from the smooth. The right hand panel show the daily change in drift rate (cm/s) over the same period. The title of each plot is the individual seal id. (TIF) [file pone.0103072.s005.tif]

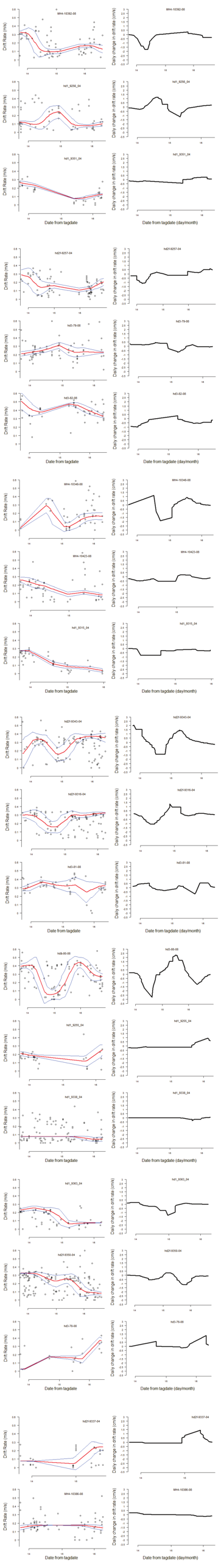

Supplement: Figure S6 — All individual females tagged in March (n = 20). Left hand panels show the fluctuation in drift rate (m/s), fitted with a smooth line. Blue lines represent 1 standard error from the smooth. The right hand panels show the daily change in drift rate (cm/s) over the same period. The title of each plot is the individual seal id. (TIF) [file pone.0103072.s006.tif]

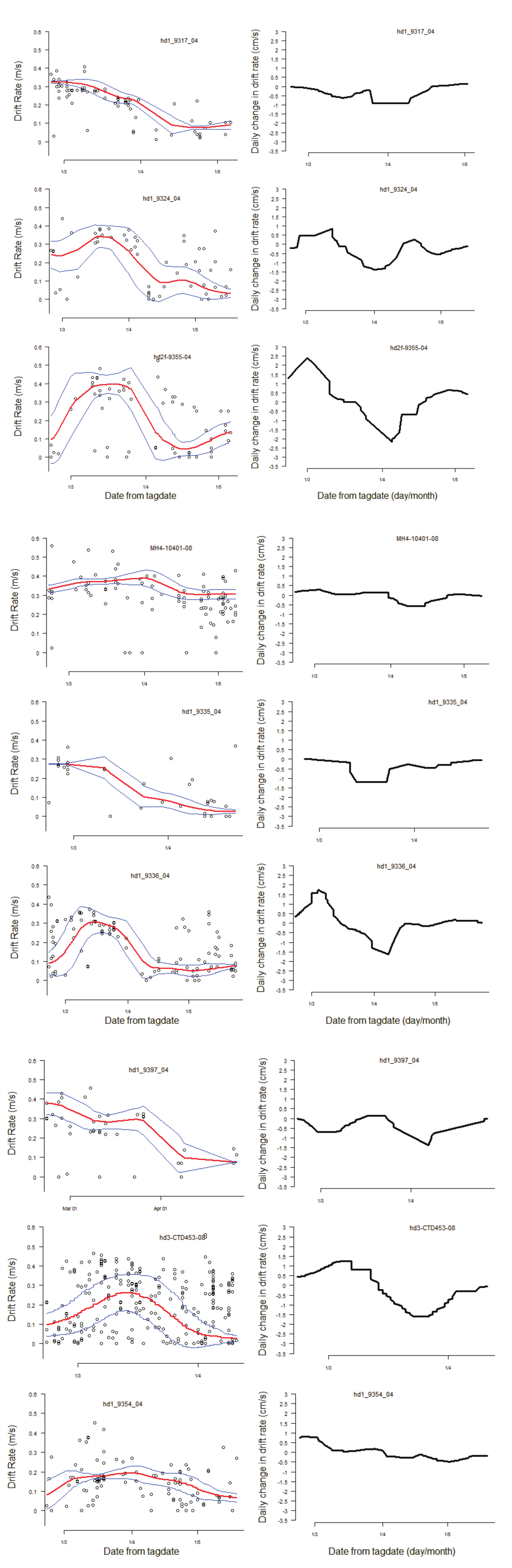

Supplement: Figure S7 — All individual males tagged in March (n = 9). Left hand panels show the fluctuation in drift rate (m/s), fitted with a smooth line. Blue lines represent 1 standard error from the smooth. The right hand panels show the daily change in drift rate (cm/s) over the same period. The title of each plot is the individual seal id. (TIF) [file pone.0103072.s007.tif]
