# Supplementary material for: Drift Diving by Hooded Seals (Cystophora cristata) in the Northwest Atlantic Ocean
Source: PLoS One. 2014 Jul 22;9(7):e103072. doi: 10.1371/journal.pone.0103072 (PMC4106908; doi:10.1371/journal.pone.0103072)
Supplement: Table S1 — Tagging information for the 47 seals. ID = individual tag numbers, Sex = males (M) or females (F), Wt = weight in kg at the time of tagging, Start = tag date, End = date when tag stopped transmitting, Days = number of days of transmissions, Latitude and Longitude = tag location. (DOC) [file pone.0103072.s008.doc]

| sort | ID | Sex | Wt (kg) | Year | Start | End | Days | Latitude | Longitude |
| --- | --- | --- | --- | --- | --- | --- | --- | --- | --- |
| 1 | hd1_9315_04 | F | 330 | 2004 | 14.3. | 31.5. | 78 | 46°58 | -62°40 |
| 2 | hd1_9256_04 | F | 208 | 2004 | 14.3. | 9.7. | 117 | 46°64 | -62°25 |
| 3 | hd1_9338_04 | F | 195.5 | 2004 | 14.3. | 13.6. | 90 | 46°63 | -62°24 |
| 4 | hd1_9255_04 | F | 276 | 2004 | 17.3. | 17.6. | 92 | 46°62 | -61°85 |
| 5 | hd2f-9257-04 | F | 148 | 2004 | 20.3. | 30.6. | 103 | 51°78 | -55°52 |
| 6 | hd2f-9337-04 | F | 150 | 2004 | 20.3. | 24.5. | 66 | 51°77 | -55°52 |
| 7 | hd2f-9350-04 | F | 182.5 | 2004 | 20.3. | 23.6. | 95 | 51°77 | -55°52 |
| 8 | hd2f-9343-04 | F | 162 | 2004 | 23.3. | 17.6. | 86 | 52°08 | -55°15 |
| 9 | hd2f-9316-04 | F | 147 | 2004 | 23.3. | 28.6. | 97 | 52°07 | -55°16 |
| 10 | hd2g-9409-04 | F | 116 | 2004 | 24.7. | 28.6. | 340 | 66°23 | -34°28 |
| 11 | hd1_9363_04 | F | 228 | 2005 | 13.3. | 6.6. | 86 | 48°03 | -61°91 |
| 12 | hd1_9351_04 | F | 188 | 2005 | 13.3. | 23.6. | 102 | 47°92 | -61°99 |
| 13 | hd5g-9400-05 | F | 112 | 2005 | 20.7. | 20.5. | 304 | 65°44 | -36°29 |
| 14 | hd5g-9420-05 | F | 138 | 2005 | 20.7. | 13.6. | 328 | 65°51 | -36°37 |
| 15 | hd5g-9413-05 | F | 90 | 2005 | 23.7. | 27.6. | 338 | 65°49 | -37°09 |
| 16 | hd5g-9344-05 | F | 108 | 2005 | 24.7. | 25.8. | 33 | 65°40 | -36°64 |
| 17 | hd5g-10222-05 | F | 98 | 2005 | 25.7. | 18.6. | 328 | 65°44 | -37°14 |
| 18 | hd5g-10227-05 | F | 114 | 2005 | 25.7. | 26.5. | 305 | 65°46 | -37°39 |
| 19 | hd5g-10206-05 | F | 95 | 2005 | 25.7. | 4.7. | 343 | 65°50 | -37°85 |
| 20 | hd5g-10205-05 | F | 138 | 2005 | 25.7. | 14.4. | 263 | 65°38 | -37°57 |
| 21 | hd6-D-06 | F | 73.5 | 2007 | 20.7. | 2.6. | 319 | 65°36 | -37°25 |
| 22 | MH4-10392-08 | F | 182.5 | 2008 | 14.3. | 18.6. | 96 | 47°69 | -61°84 |
| 23 | MH4-10423-08 | F | 251 | 2008 | 14.3. | 1.7. | 109 | 47°68 | -61°83 |
| 24 | MH4-10348-08 | F | 251 | 2008 | 15.3. | 26.6. | 104 | 47°69 | -61°81 |
| 25 | MH4-10386-08 | F | 224.5 | 2008 | 15.3. | 14.6. | 91 | 47°69 | -61°77 |
| 26 | hd3-80-08 | F | 155.5 | 2008 | 24.3. | 11.7. | 109 | 49°87 | -52°32 |
| 27 | hd3-81-08 | F | 158.5 | 2008 | 24.3. | 2.7. | 100 | 49°69 | -52°16 |
| 28 | hd3-82-08 | F | 139 | 2008 | 24.3. | 23.6. | 90 | 49°72 | -52°18 |
| 29 | hd3-79-08 | F | 149.5 | 2008 | 25.3. | 19.6. | 86 | 49°65 | -52°30 |
| 30 | hd3-78-08 | F | 229 | 2008 | 25.3. | 21.6. | 88 | 49°96 | -51°87 |
| 31 | hd1_9324_04 | M | 321 | 2004 | 16.3. | 15.6. | 91 | 46°60 | -61°85 |
| 32 | hd1_9335_04 | M | 326.5 | 2004 | 17.3. | 8.6. | 83 | 46°64 | -61°87 |
| 33 | hd1_9336_04 | M | 192 | 2004 | 17.3. | 21.7. | 126 | 46°57 | -61°82 |
| 34 | hd1_9317_04 | M | 274 | 2004 | 19.3. | 14.6. | 87 | 46°47 | -61°90 |
| 35 | hd2f-9355-04 | M | 246 | 2004 | 25.3. | 24.6. | 92 | 51°87 | -55°40 |
| 36 | hd2g-9421-04 | M | 172 | 2004 | 24.7. | 20.3. | 239 | 66°20 | -33°48 |
| 37 | hd1_9397_04 | M | 338 | 2005 | 12.3. | 16.6. | 95 | 47°98 | -61°84 |
| 38 | hd1_9341_04 | M | 338.5 | 2005 | 14.3. | 3.7. | 111 | 47°77 | -61°99 |
| 39 | hd5g-9427-05 | M | 194 | 2005 | 20.7. | 7.4. | 261 | 65°50 | -36°02 |
| 40 | hd5g-10204-05 | M | 146 | 2005 | 24.7. | 13.6. | 324 | 65°23 | -36°83 |
| 41 | hd5g-10207-05 | M | 174 | 2005 | 24.7. | 12.7. | 353 | 65°46 | -37°23 |
| 42 | hd5g-10188-05 | M | 109 | 2005 | 25.7. | 22.3. | 240 | 65°40 | -37°46 |
| 43 | hd6-F-06 | M | 97.5 | 2007 | 24.7. | 24.6. | 336 | 65°40 | -37°82 |
| 44 | ct18-L-06 | M | 130 | 2007 | 24.7. | 7.4. | 258 | 65°38 | -37°97 |
| 45 | MH4-10401-08 | M | 352.5 | 2008 | 16.3. | 22.6. | 98 | 47°66 | -61°76 |
| 46 | hd3-CTD453-08 | M | 230 | 2008 | 24.3. | 14.5. | 51 | 49°66 | -52°62 |
| 47 | hd1_9354_04 | M | 301 | 2005 | 12.3. | 23.6. | 103 | 47°98 | -61°84 |
