# Supplementary material for: Drift Diving by Hooded Seals (Cystophora cristata) in the Northwest Atlantic Ocean
Source: PLoS One. 2014 Jul 22;9(7):e103072. doi: 10.1371/journal.pone.0103072 (PMC4106908; doi:10.1371/journal.pone.0103072)
Supplement: Table S2 — AIC table. The response variable, daily change in drift rate, was investigated in relation to geographic location and day of year. Loglik is the loglikelihood, K is the number of parameters in the model. AICi is AIC for model i, and ΔAIC is the difference between the AIC of the best fitting model and that of model i. Exp(−0.5Δi) represent the relative likelihoods and the w i is the Akiake weights. D.E% is the deviance explained by the model. Models with and without sex as an interaction term was run. (DOC) [file pone.0103072.s009.doc]

| **model** | **loglik** | **K** | **AIC** | **ΔAIC** | **exp(0.5Δ_i_)** | ***w_i_*** | **DE(%)** |
| --- | --- | --- | --- | --- | --- | --- | --- |
| Geographic location and day of year, by sex | -3876.8 | **5** | 7763.59 | 0 | 1 | 1 | **27** |
| Geographic location, by sex | -4197.61 | 3 | 8401.23 | 637.64 | 3.4602E-139 | 3.4602E-139 | 19.8 |
| Geographic loction and day of year | -4368.03 | 3 | 8742.06 | 978.47 | 3.3724E-213 | 3.3724E-213 | 15.7 |
| Day of year, by sex | -4457.23 | 3 | 8920.46 | 1156.87 | 6.1438E-252 | 6.1438E-252 | 13.4 |
| Geographic location | -4560.29 | 2 | 9124.58 | 1360.99 | 2.9159E-296 | 2.9159E-296 | 10.8 |
| Day of year | -4611.98 | 2 | 9227.97 | 1464.38 | 0 | 0 | 9.4 |
